# The pioneer and differentiation factor FOXA2 is a key driver of yolk‐sac tumour formation and a new biomarker for paediatric and adult yolk‐sac tumours

**DOI:** 10.1111/jcmm.16222

**Published:** 2021-01-14

**Authors:** Wasco Wruck, Felix Bremmer, Mara Kotthoff, Alexander Fichtner, Margaretha A. Skowron, Stefan Schönberger, Gabriele Calaminus, Christian Vokuhl, David Pfister, Axel Heidenreich, Peter Albers, James Adjaye, Daniel Nettersheim

**Affiliations:** ^1^ Institute for Stem Cell Research and Regenerative Medicine University Hospital Düsseldorf Düsseldorf Germany; ^2^ Institute of Pathology University Medical Center Goettingen Goettingen Germany; ^3^ Department of Urology Urological Research Lab Translational UroOncology University Hospital Düsseldorf Düsseldorf Germany; ^4^ Department of Pediatric Hematology and Oncology University Children's Hospital Essen Germany; ^5^ Department of Pediatric Hematology and Oncology University Hospital Bonn Bonn Germany; ^6^ Institute of Pathology University Hospital Bonn Bonn Germany; ^7^ Department of Urology University Hospital Cologne Cologne Germany; ^8^ Department of Urology University Hospital Düsseldorf Düsseldorf Germany

**Keywords:** adult and paediatric germ cell tumours, biomarker, DNA methylation, embryonal carcinomas, FOXA2, microRNA, Pluripotency, SOX17, Yolk‐sac tumours

## Abstract

Yolk‐sac tumours (YSTs), a germ cell tumour subtype, occur in newborns and infants as well as in young adults of age 14‐44 years. In clinics, adult patients with YSTs face a poor prognosis, as these tumours are often therapy‐resistant and count for many germ cell tumour related deaths. So far, the molecular and (epi)genetic mechanisms that control development of YST are far from being understood. We deciphered the molecular and (epi)genetic mechanisms regulating YST formation by meta‐analysing high‐throughput data of gene and microRNA expression, DNA methylation and mutational burden. We validated our findings by qRT‐PCR and immunohistochemical analyses of paediatric and adult YSTs. On a molecular level, paediatric and adult YSTs were nearly indistinguishable, but were considerably different from embryonal carcinomas, the stem cell precursor of YSTs. We identified *FOXA2* as a putative key driver of YST formation, subsequently inducing *AFP*, *GPC3*, *APOA1/APOB*, *ALB* and *GATA3/4/6* expression. In YSTs, WNT‐, BMP‐ and MAPK signalling‐related genes were up‐regulated, while pluripotency‐ and (primordial) germ cell‐associated genes were down‐regulated. Expression of *FOXA2* and related key factors seems to be regulated by DNA methylation, histone methylation / acetylation and microRNAs. Additionally, our results highlight FOXA2 as a promising new biomarker for paediatric and adult YSTs.

## 1 INTRODUCTION

Testicular type II germ cell tumours (GCTs) represent the most common tumour of young men of age 17‐45 years and incidence rates are rising steadily.^1^, ^2^ Type II GCTs can be stratified into seminomas and non‐seminomas, which both arise from the precursor lesion germ cell neoplasia in situ (GCNIS) as a result of a defective primordial germ cell (PGC) development.^1^, ^3^ Seminomas are highly similar to GCNIS and PGCs with regard to morphology, gene expression and epigenetics.^1^ The non‐seminomas have their own stem cell population—the embryonal carcinoma (EC).^1^ ECs are pluri‐ to totipotent and able to differentiate into cells of all three germ layers, resulting in formation of teratomas, and into extra‐embryonic tissues, that is yolk‐sac tumours (YSTs) and choriocarcinomas.^1^ In clinics, patients with YSTs face a poor prognosis, as YSTs count for many GCT‐related deaths. YSTs frequently develop resistance towards the standard cisplatin‐based therapy and cannot be cured by current standard treatment protocols. GCTs can also be found in newborns and infants, where these tumours are termed type I GCTs, which do not develop from GCNIS, but from an early defective PGC.^1^ They present mainly as teratomas and YSTs. Thus, in paediatric GCTs, occurrence and treatability of YSTs are also important issues.

The molecular and (epi)genetic mechanisms that control differentiation of ECs into YST are still unclear. In a previous study, we demonstrated that in vivo reprogramming of seminoma cells (TCam‐2) into a non‐seminoma‐like cell fate (EC) can be induced by inhibiting the BMP‐pathway.^4^ We identified SOX2, which was strongly up‐regulated in response to BMP‐pathway inhibition, as the key effector of this reprogramming process.^5^ In nude mice, TCam‐2 cells deficient for SOX2 were not able to reprogramme to an EC anymore, but induced differentiation into YST‐like tissues instead. Further analyses demonstrated that FOXA2, a pioneer and differentiation factor, might be the key driver of this YST‐like differentiation, as FOXA2 interacts with typical YST‐associated factors like AFP, APOA1, ALB and HAND1.^5^ Consequently, TCam‐2 cells deficient for SOX2 and FOXA2 were not able to differentiate into non‐seminoma‐like cells at all.^6^ Thus, we suggest that FOXA2 might be the key effector in development of YSTs.

## 2 MATERIAL AND METHODS

### 2.1 Cell culture

All GCT cell lines were cultivated as described previously.^7^ See Table S1 for detailed information on cell lines (Table S1). STR profiles of all cell lines are checked on a regular basis and are available upon request.

### 2.2 RNA isolation

RNA was isolated from cell lines using the RNAeasy Mini Kit (Qiagen, Hilden, Germany) according to the manufacturer's protocol. RNA from frozen type I YST tissues was isolated by TRIzol reagent according to the manual (Qiagen).

### 2.3 Quantitative RT‐PCR

cDNA synthesis and quantitative reverse transcription‐polymerase chain reaction (qRT‐PCR) were performed as described previously.^7^ Briefly, 1µg of total RNA was in vitro transcribed into cDNA. Each sample was analysed in technical triplicates using 7.34 ng cDNA for each replicate. Oligonucleotide sequences are given in Table S2.

### 2.4 Immunohistochemistry

Immunohistochemistry (IHC) was performed as published previously.^8^ Briefly, antigen retrieval was carried out in citrate buffer. The primary antibodies were incubated for 30 minutes (min) at room temperature. Sections were incubated with a ready‐to‐use HRP‐labelled secondary antibody at RT for 25 min. The substrate DAB + Chromogen system was used to visualize the antigen. Tissues were counterstained with Meyer's haematoxylin. For antibody details, see Table S3.

### 2.5 Meta analyses of expression, DNA methylation and microRNA data

2.5.1

Affymetrix expression array raw data (CEL files) of paediatric and adult YSTs as well as ECs and human embryonic stem cells were read into the R/Bioconductor environment using the package ‘affy’.^9^, ^10^ Data were transformed to the logarithmic (base 2) scale and normalized with the ‘Robust Multi‐array average’ (RMA) method. Detection *P*‐values were determined with the ‘MAS5’ method implemented in the function ‘*mas5calls’* from the package ‘affy’. Genes with detection *P*‐values ≤ 0.05 were considered to be expressed. Pearson correlation coefficients between samples were calculated with the R‐built‐in function ‘*cor’*. Principal component analysis (PCA) of the samples was achieved through the R‐built‐in function ‘*prcomp’,* while for cluster analysis the R package ‘dendextend’ was employed.^11^ For identification of differentially expressed genes, linear models for microarrays as provided by the Bioconductor ‘limma’ package were fitted to the data.^12^ The p‐values resulting from the ‘limma’ test were adjusted for the false discovery rate via the ‘qvalue’ package.^13^ Differentially expressed genes were assessed using the following criteria: detection *P*‐value ≤ 0.05 at least in YSTs, limma‐q‐value ≤ 0.05, ratio ≥ 1.33 (up‐regulation in YSTs); detection *P*‐value ≤ 0.05 at least in ECs, limma‐q‐value ≤ 0.05, ratio ≤ 0.75 (down‐regulation in YSTs).

#### 2.5.2 Meta‐analysis—DNA methylation

Methylation data sets of human adult YSTs and ECs were downloaded from ‘The Cancer Genome Atlas’ (TCGA).^14^ Pre‐processed DNA methylation data were used, and YSTs and EC samples were extracted from the testicular cancer data set. Beta‐values provided in the pre‐processed DNA methylation data were converted to M‐values using the formula suggested by Du et al^15^:

$$M=\log_{2}\left(\frac{\text{beta}}{1‐\text{beta}}\right)$$

Quality control procedures included Pearson correlation coefficients as described above and cluster dendrograms via the R package ‘dendextend’.^11^ Differential DNA methylation was determined based on M‐values using the test from the Bioconductor ‘limma’ package and the ‘qvalue’ package for p‐value adjustment. Differentially higher DNA methylation in YSTs was determined by the criteria: M ≥ 2 (in YSTs) or M ≤ ‐2 (in ECs), log_ratio_ ≥ 2 (log ratio = M_YST_ – M_EC_) and limma‐q‐value ≤ 0.05. Differentially lower DNA methylation in YSTs was determined by the criteria: M ≥ 2 (in ECs) or M ≤ ‐2 (in YSTs), log_ratio_ ≤ ‐2 and limma‐q‐value ≤ 0.05.

#### 2.5.3 Meta‐analysis—microRNAs

Analogously to DNA methylation data, microRNA data sets of human adult YSTs and ECs were downloaded from TCGA. Pre‐processed microRNA data providing RPKM ( reads per kilobase exon per million reads) values were used, and YST and EC samples were extracted from the TGCT data set. For determination of differential expression, a detection level of RPKM ≥ 1 (for up‐regulation in YSTs, for down‐regulation in ECs) was used together with a ratio ≥ 2 (up‐regulation) or ratio ≤ 0.5 (down‐regulation) and a limma‐q‐value ≤ 0.05. MicroRNA targets (homo sapiens) were downloaded from the TargetScan 7.2 database.^16^

### 2.6 Analysis tools

Venn diagrams were generated using Venny 2.1.^17^ The STRING algorithm was used to predict interactions between genes/proteins by confidence and to search for genes/proteins grouping to various sub‐categories of the ‘Gene Ontology’ (GO) category ‘biological processes’.^18^ TCGA data sets were analysed using cBioPortal.^14^, ^19^ Histone‐chromatin‐immunoprecipitation‐sequencing (Histone‐ChIP‐seq) data sets were extracted from the ‘Encyclopedia of DNA elements’ (ENCODE) project and analysed via the UCSC Genome Browser.^20^, ^21^, ^22^ FOXA2 target genes were extracted from the ‘Harmonizome’ database.^23^

## 3 RESULTS

To identify factors that are involved in YST formation, we meta‐analysed gene expression microarray data of paediatric YSTs (pYST), adult YSTs (aYST) and ECs as well as human embryonic stem cells (hESC) as controls^24^, ^25^, ^26^, ^27^, ^28^ (Data S1A‐E). First, we compared the gene expression profiles of all samples to each other using a correlation matrix, unsupervised hierarchical clustering and a principle component analysis, demonstrating a high similarity of pYST to aYSTs (YST cluster) and among EC samples (EC cluster) (Figure S1A–C). The EC samples grouped with the hESCs and clearly apart from the p/aYSTs, while pYSTs and aYSTs were highly similar to each other (Figure S1A–C).

Next, we identified all genes differentially expressed between aYSTs and ECs (Data S1B). We found 126 individual genes up‐regulated and 186 down‐regulated in aYSTs compared with ECs (fold change (FC) >4) (Data S1C).

**FIGURE 1:**
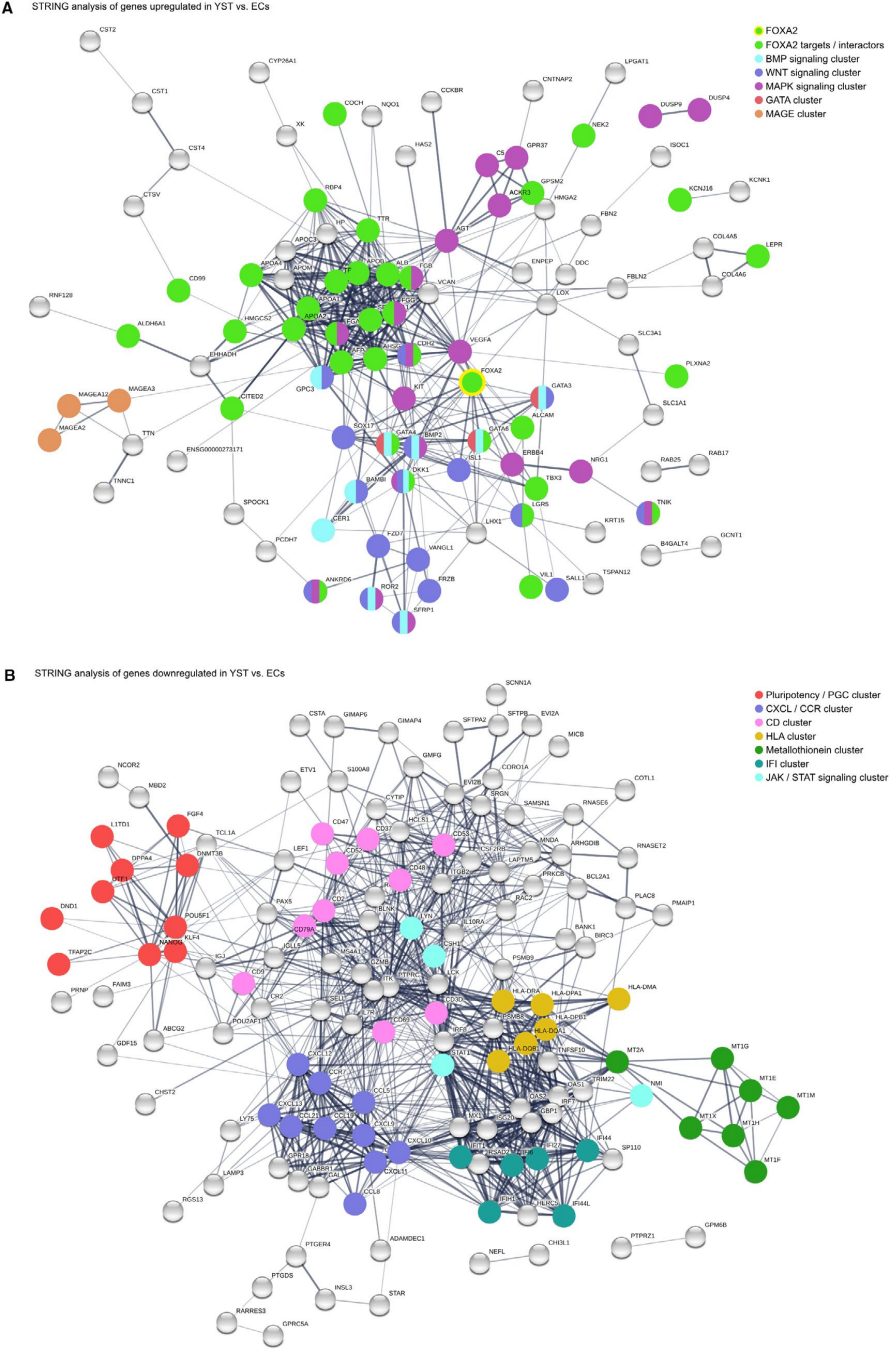
(A, B) STRING‐based interaction prediction of genes up‐regulated (A) or down‐regulated (B) in aYST versus ECs. FOXA2 target genes are highlighted in green (A) according to the ‘Harmonizome’ database.^23^ Genes belonging to the same ‘biological process’ GO category were labelled by colours as indicated

By using the STRING algorithm combined with a Gene Ontology (GO) search, we predicted interactions and the involved biological processes of up‐ and down‐regulated genes (Figure 1A). Among the genes up‐regulated in aYST versus ECs, we found *FOXA2* and *SOX17* as well as many FOXA2‐related genes, like *AFP*, *GPC3*, *APOA1/A2/B*, *ALB*, *TTR*, *FGA/B/G* and *DKK1* ^5^, ^6^, ^23^ (Figure 1A, green labelled). Furthermore, interaction of *GATA* differentiation factors (*GATA3/4/6*; red labelled), WNT signalling‐related (*ANKRD6*, *BAMBI*, *BMP2*, *CDH2*, *DKK1*, *FRZB*, *FZD7*, *GATA3*, *GPC3*, *ISL1*, *LGR5*, *ROR2*, *SALL1*, *SFRP1*, *SOX17*, *TNIK*, *VANGL1*; blue labelled), BMP signalling‐related (*BAMBI*, *BMP2*, *CER1*, *DKK1*, *GATA3/4/6*, *GPC3*, *ROR2*, *SFRP1*; turquoise labelled) and MAPK signalling‐related factors (*ACKR3*, *AGT*, *ANKRD6*, *BMP2*, *C5*, *CDH2*, *DKK1*, *DUSP4/9*, *ERBB4*, *FGA/B/G*, *FZD7*, *GPR37*, *KIT*, *NRG1*, *ROR2*, *SFRP1*, *TNIK*, *VEGFA*; purple labelled) was predicted in aYSTs. Additionally, three ‘Cancer / Testis‐Antigen’ (CTA) members of the MAGE family were up‐regulated (*MAGEA2/3/12*; orange labelled).

In contrast, in aYSTs we found strong down‐regulation of pluripotency / EC / PGC‐related factors, such as *NANOG*, *OCT3/4*, *KLF4*, *DPPA4*, *UTF1*, *FGF4*, *DNMT3B*, *L1TD1*, *TFAP2C* and *DND1* (Figure 1B, red labelled). Additionally, metallothioneins (Figure 1B, green labelled), HLA molecules (*HLA‐DPA1/B*, ‐*DQA1/B1*, ‐*DRA* ‐*DMA*) (Figure 1B, yellow labelled), CXCL and chemokine factors (*CXCL9/10/11/12/13; CCR7*, *CCL5/8/19/21*) (Figure 1B, blue labelled), ‘cluster of differentiation’ (CD) genes (*CD2/3D/9/37/47/48/52/53/69/79A)* (Figure 1B, pink labelled) and ‘interferon inducible proteins’ (IFI6/27/44/44L/H1/T1) (Figure 1B, patrol labelled) were down‐regulated in aYST compared with ECs.

To find differences between pYSTs and aYSTs, we screened for differentially expressed genes (Data S1D) and identified only 19 genes (FC > 4; 12 up‐regulated, 7 down‐regulated in aYSTs vs. pYSTs) (Data S1E). Among them, *MAGEA2*/*A3*/*A12* and cell cycle‐related genes *CCND2* (up‐regulated in aYSTs) and *CDKN1C* (down‐regulated in aYSTs) (Data S1E). Thus, pYST and aYST are highly similar with regard to gene expression.

**FIGURE 2:**
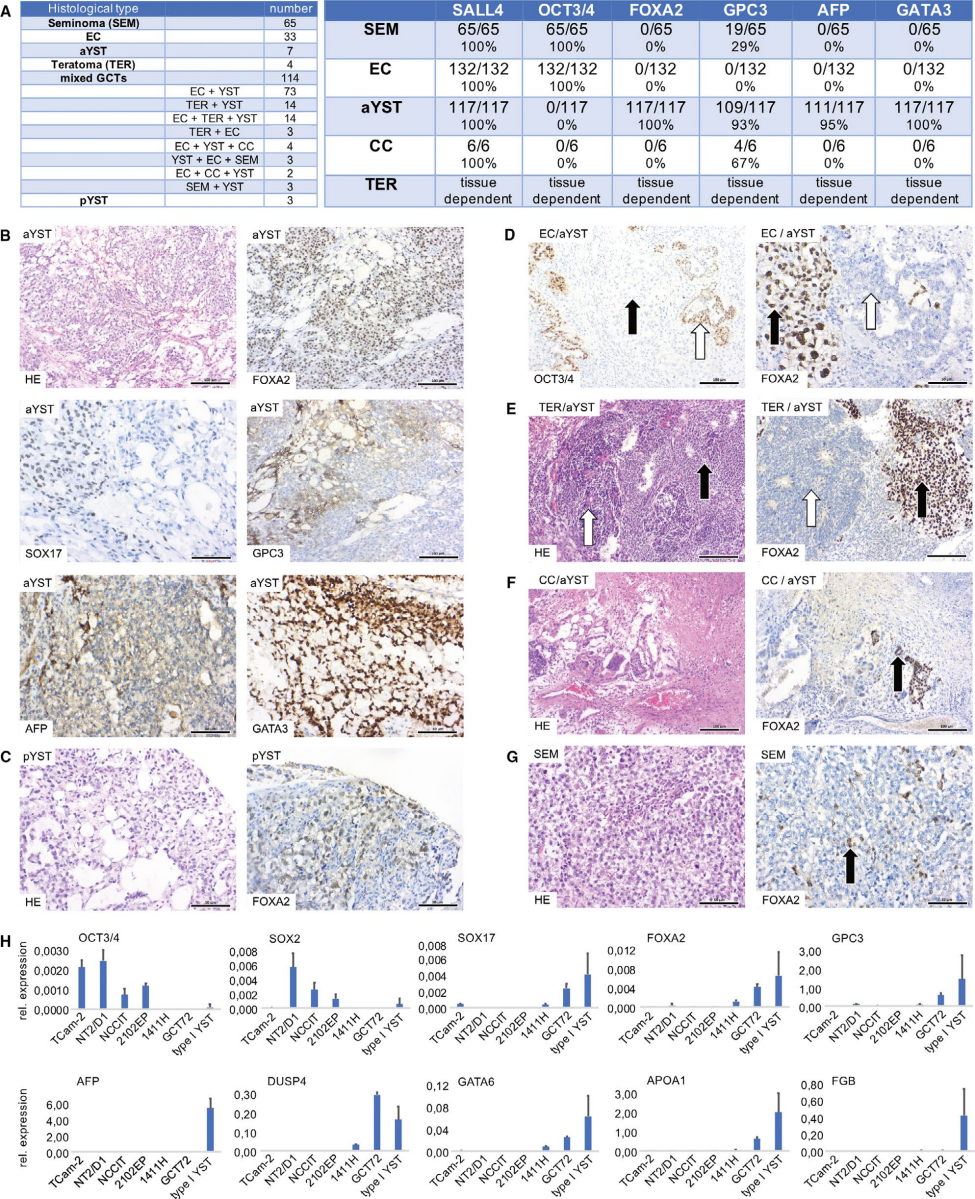
(A) Table summarizing numbers of analysed GCT tissues based on histology (left) and evaluation of IHC stainings (right). (B) HE staining and IHC for FOXA2, SOX17, GPC3, AFP and GATA3 in aYST tissues. (C) HE staining and FOXA2‐IHC in pYSTs. (D) OCT3/4‐ and FOXA2‐IHC in mixed tumours with EC (white arrows) and aYST (black arrows) components. (E) HE staining and FOXA2‐IHC in mixed tumours with teratoma (TER; white arrows) and aYST (black arrows) components. (F) HE staining and FOXA2‐IHC in mixed tumours with choriocarcinoma (CC; white arrows) and aYST (black arrows) components. (G) HE staining and FOXA2‐IHC in a seminoma (SEM) patient with elevated serum AFP levels. FOXA2‐positive aYST cells could be found (black arrow). (B‐G) Scale bars: 50/100 µm. (D) qRT‐PCR analysis of pluripotency and YST marker genes in pYSTs (n = 6), seminoma cells (TCam‐2), EC cells (2102EP, NCCIT, NT2/D1), EC‐YST cells (1411H) and aYST cells (GCT72). *GAPDH* and *ACTB* were used as housekeepers and for normalization

We verified results by IHC on 342 FFPE‐GCT‐tissues (Figure 2A). We stained all samples for SALL4, OCT3/4, FOXA2, SOX17, GPC3, AFP and GATA3 (Figure 2A). All examined aYST populations (n = 117) showed a strong nuclear expression of FOXA2 (100%) and GATA3 (100%), while being focally positive for SOX17 (100%), GPC3 (93%) and AFP (95%) (Figure 2A,B). pYSTs were also positive for FOXA2 (100%) (Figure 2C). In mixed GCTs, FOXA2 clearly distinguished EC (Figure 2D, white arrows), teratoma (Figure 2E, white arrows) and choriocarcinoma (Figure 2F, white arrows) components from YST populations (Figure 2D‐F, black arrows). All analysed tissues were positive for the GCT marker SALL4 (Figure 2A).^29^, ^30^ We also analysed three seminoma patients showing increased levels of serum AFP. In these samples, FOXA2‐positive cells could be detected, demonstrating presence of a YST component (Figure 2G, black arrow). Taken together, FOXA2 presents as a new and highly specific biomarker for p/aYSTs and is able to detect single YST cells in mixed GCTs. FOXA2 might also be a valuable biomarker to detect occult YSTs, e. g. in seminomas with elevated AFP levels.

By qRT‐PCR, we analysed expression of pluripotency and YST marker genes in pYSTs (n = 6), the seminoma cell line TCam‐2, three EC cell lines (2102EP, NCCIT, NT2/D1), an EC‐aYST cell line (1411H) and an aYST cell line (GCT72) (Figure 2H). 1411H growths as an EC in vitro, but differentiates into YST upon xenotransplantation into nude mice.^31^ Thus, in vitro 1411H resembles an intermediate between EC and aYST. We found negligible expression of the pluripotency factors *OCT3/4* and *SOX2* in pYSTs, 1411H and GCT27 compared withthe EC / seminoma cell lines, while the proposed aYST key factors *FOXA2*, *SOX17*, *GPC3*, *DUSP4, AFP, GATA6, APOA1 and FGB* were highly expressed in pYSTs (Figure 2H). Compared with the seminoma / EC cell lines, the aYST cell line GCT72 showed high expression of the proposed YST factors (except *AFP* and *FGB*), respectively (Figure 2H).

Next, we screened the TCGA ‘Testicular GCT’ cohort for gene expression and the mutational burden of the YST key factors (Figure S2A,B). We included pure YSTs, mixed GCTs with YST component, ECs and seminomas (Figure S2A,B). All samples were isolated from testes, harbour the 12p gain, were highly aneuploid and mainly in a GCT‐typical age of 14 ‐ 44 (Figure S2A).

Mutations in the YST key factors were overall very rare; in total we found only 4 amplifications, 13 deletions, 3 missense mutations and 1 truncation (Figure S2A). The mutation frequency was below 1%, only SOX17 (4%), GATA3 (2.1%) and APOA1 (1.4%) showed a slightly increased frequency (Figure S2A). Pure ECs and aYSTs showed no mutations in analysed genes (Figure S2A). Thus, mutations of YST key factors play no role in YST formation or aggressiveness.

In contrast, expression of proposed YST key factors could clearly be associated with YST tissues and YST‐containing mixed GCTs, but not with ECs or seminomas (except *SOX17* and *GATA4*) (Figure S2B). Thus, we could confirm our hypothesis that expression of proposed key factors is linked to a YST cell fate.

We asked how aYSTs differ from ECs with respect to DNA methylation and microRNA expression. We performed an unsupervised hierarchical clustering of DNA methylation and microRNA data of all aYST (n = 3) and pure EC (n = 25) samples of the TCGA cohort, demonstrating that aYSTs and ECs can be stratified based on DNA methylation and microRNA expression (Figure S1D,E).

**FIGURE 3:**
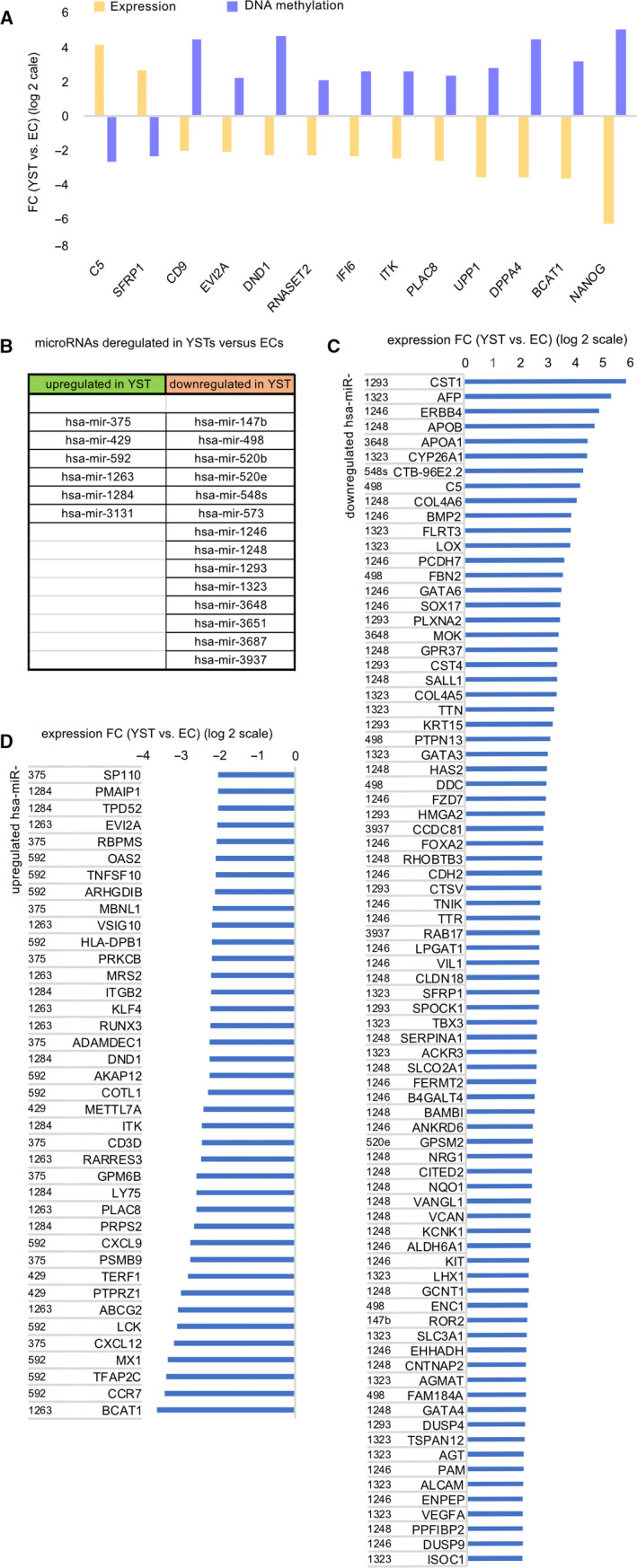
(A) Genes deregulated (FC ≥ 4) in aYSTs versus ECs and showing inverse correlation to DNA methylation. (B) Deregulated microRNAs in aYSTs versus ECs. (C, D) Waterfall diagrams of expression dynamics (FC ≥ 4) of putative target genes of indicated microRNAs

We correlated DNA methylation to gene expression to identify genes putatively regulated by DNA methylation. Genes showing an inverse correlation of DNA methylation to expression were of highest interest. Of all genes significantly down‐regulated in aYSTs versus ECs (FC > 4), only 2 correlated with increasing DNA methylation levels (aYST vs. EC) (Group 1); of all genes up‐regulated in aYST versus ECs (FC > 4), 11 correlated with decreasing DNA methylation levels (Group 2) (Figure 3A) (Data S1F). Thus, Group 1 genes represent factors expressed in ECs and becoming down‐regulated and hypermethylated in YSTs. Group 2 genes represent factors silenced in expression by DNA methylation in ECs and induced and hypomethylated upon YST formation.

We screened for microRNAs differentially expressed between aYSTs and ECs and showing an inverse correlation with gene expression of related targets (identified by TargetScan 1.7) (Data S1G). We identified 6 microRNAs that were significantly up‐regulated in aYSTs versus ECs and could be linked to 39 target genes down‐regulated in YSTs (FC > 4) (Group 1; Figure 3B,C), while 14 down‐regulated microRNAs could be linked to 80 genes up‐regulated in YSTs (FC > 4) (Group 2) (Figure 3B,D). Thus, Group 1 represents microRNAs up‐regulated during YST formation and involved in repressing the pluripotency and PGC programme (*KLF4*, *BCAT1*, *DND1*, *TFAP2C*) as well as chemokine signalling (*CCR7*, *CXCL9*, *CXCL12*). Vice versa, Group 2 represents microRNAs down‐regulated in YSTs and leading to de‐repression of typical YST‐associated genes, such as *FOXA2*, *SOX17*, *AFP*, *APOA1*, *APOB*, *GATA3/4/6*, *BMP2*, *BAMBI*, *FZD7* and *DUSP4/9*.

Our results suggest that expression of *FOXA2*, which we postulate as a p/aYST key factor, is not regulated by DNA methylation, but might be repressed in ECs by *microRNA1246* (Figure 3C). Furthermore, we asked, if *FOXA2* expression might be regulated by epigenetic modifications on histone level. Thus, we screened ChiP‐seq data extracted from the ENCODE project of various activating or repressing histone marks throughout the *FOXA2* genomic locus in NT2/D1 EC and HepG2 hepatocellular carcinoma cells (Figure S3). HepG2 cells strongly express *FOXA2* and many factors also found up‐regulated during YST formation (*AFP*, *APOA1/A2/B*, *ALB*, *FGA/B/G, GATA3/4/6*, etc.; Figure S2B) and thus represent a valuable proxy for studying FOXA2 interactions.^32^, ^33^ In *FOXA2*‐ NT2/D1 cells, we found high levels of repressive H3K27me3 mark around the *FOXA2* transcription start site (red labelled) and throughout the gene body, while *FOXA2*+  HepG2 cells harboured high levels of activating H3K4me1/me3, H3K9ac and transcription promoting H3K36me3 (Figure S3). Thus, we propose that in ECs *FOXA2* expression is silenced by *microRNA1246* and repressive epigenetic modifications, for example H3K27me3, which need to be removed to allow for expression during differentiation. Additionally, induction of *FOXA2* in p/aYSTs might be accompanied by activating histone modifications like H3K4me1/3, H3K9ac and H3K36me3.

## 4 DISCUSSION

In this study, we deciphered the molecular and epigenetic mechanisms that regulate formation of YSTs and highlight FOXA2 as a new biomarker for p/aYSTs.

We demonstrated that pYSTs and aYSTs are highly similar to each other with regard to gene expression, but aYSTs were clearly distinguishable from ECs based on gene and microRNA expression as well as DNA methylation. Thus, we assume that molecular mechanisms found in aYSTs are similarly detectable in pYSTs. Additionally, therapeutic options tested in aYSTs might also apply for pYSTs.

In our study, we highlight FOXA2 as a key driver of p/aYST formation acting in concert with SOX17, which already has been found up‐regulated in p/aYSTs versus seminomas.^34^ FOXA2 is a pioneer and endodermal transcription factor expressed in several tumour types including genitourinary cancers, such as bladder carcinomas and prostate cancer and playing a crucial role in cellular differentiation.^35^, ^36^, ^37^

**FIGURE 4:**
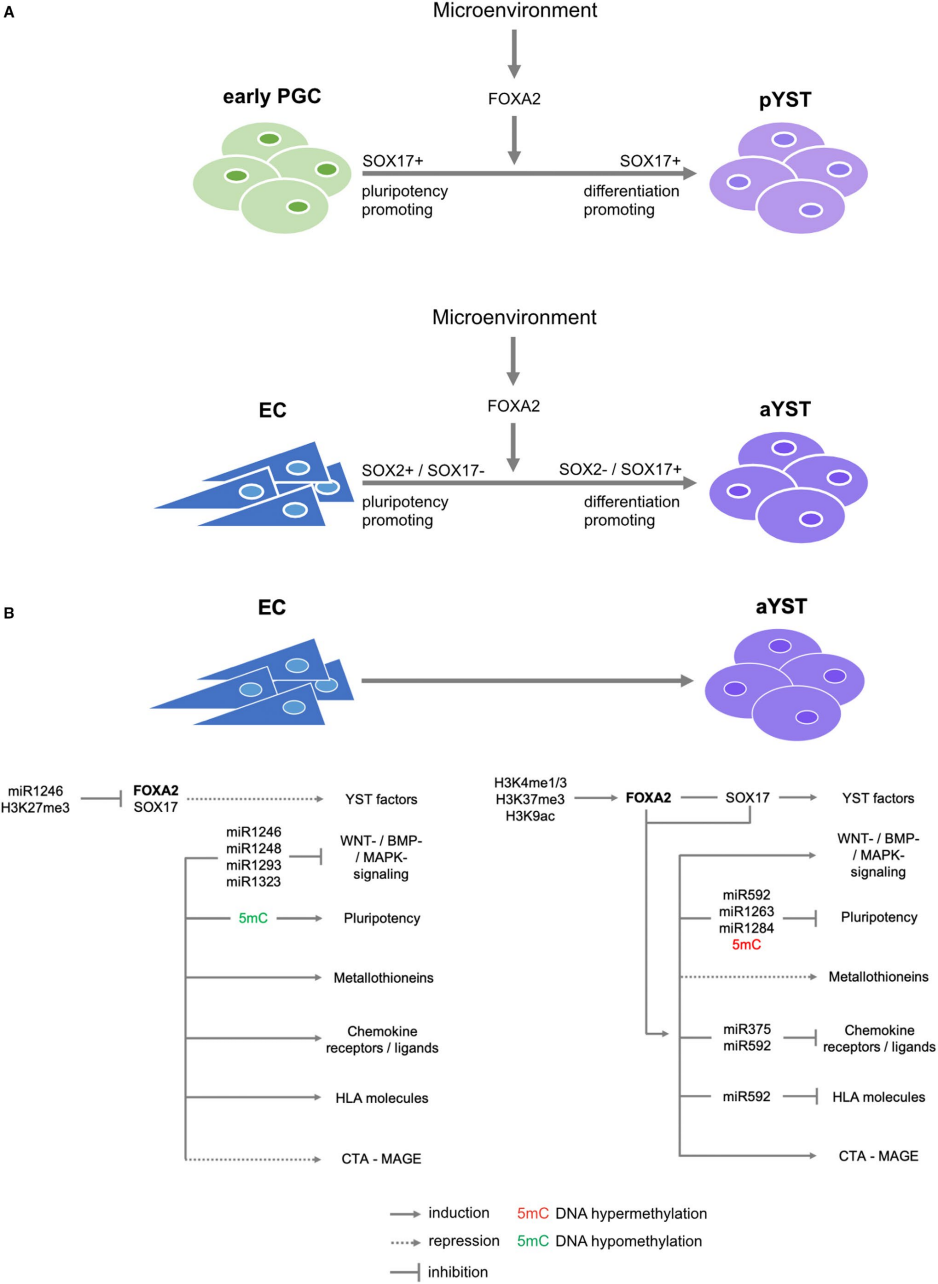
Models summarizing the findings of this study. (A) Formation of pYSTs from PGCs (type I GCT) and aYSTs from ECs (type II GCT) might be the result of a microenvironment‐triggered up‐regulation of *FOXA2*. In SOX17+ (defective) PGCs, FOXA2 and SOX17 cooperatively induce differentitaion into pYST lineage. In ECs, upregulation of*FOXA2* subsequently leads to induction of SOX17. In both, defective PGCs and ECs, upregulation of *FOXA2* causes switching of SOX17 from a pluripotency‐promoting into a differentiation‐inducing factor. (B) Model summarizing the molecular and epigenetic events influencing and regulating development of aYSTs from ECs in detail

We suggested previously that during in vivo differentiation of TCam‐2 cells into a non‐seminoma including aYST‐like structures, SOX17 switches function from pluripotency‐promoting to differentiation‐inducing as a result of FOXA2 up‐regulation.^5^, ^6^ Interestingly, partnering of SOX2 with PAX6 (instead of OCT3/4) leads to a switch in the function of SOX2 from pluripotency‐promoting to endodermal differentiation‐inducing.^38^ Given the high similarity between SOX factors and that SOX2 and SOX17 share similar functions in regulating pluripotency in ECs and seminomas, respectively, it seems reasonable that both factors are able to switch their functions in dependency of the interacting partner.^39^ The finding that SOX17 might be a key factor of YST formation strengthens the idea that pYSTs, which do not originate from a GCNIS, arise from an early SOX17+ PGC, where SOX17 switches function as a result of a microenvironment‐triggered FOXA2 induction (Figure 4A).^40^ We suggest further that in  SOX17‐/SOX2+ ECs, a microenvironment‐triggered FOXA2 induction (accompanied by inhibition of *miR1246*, removal of repressive H3K27me3 and establishment of activating H3K4me1/3, H3K9ac and H3K36me3.) is an initial step in aYST formation, leading to up‐regulation of SOX17 (Figure 4A).^41^ We propose that in ECs, seminomas and PGCs, SOX17 is able to switch from a pluripotency‐promoting to a differentiation‐inducing factor upon microenvironment‐triggered FOXA2 induction, driving differentiation into YST lineage (Figure 4A).

Our data suggest that up‐regulation of FOXA2 leads to induction of p/aYST‐associated genes *SOX17*, *AFP*, *APOA1/A2/B*, *ALB*, *TTR*, *FGA/B/G and GATA3/4/6* etc (Figure 4 B). Interestingly, expression of these factors (including *FOXA2*) is highly typical for liver cells, that is hepatocytes.^42^ We also detected high expression of these factors in hepatocellular carcinomas (of both sexes) (Figure S2B). Furthermore, the WNT and BMP pathways play an important role in differentiation of human pluripotent stem cells to hepatocytes.^42^ Interaction of FOXA2 with many of the YST‐related genes and signalling pathways has been shown in various settings including liver development and hepatocellular carcinomas.^5^, ^35^, ^43^, ^44^, ^45^, ^46^ Additionally, in stem cell context *SOX17* is an endodermal differentiation factor.^39^ Thus, on a molecular level, YST cells are closely related to liver (carcinoma) cells.

We detected up‐regulation of WNT, BMP and MAPK signalling factors in aYSTs versus ECs. In parallel, the pluripotency programme was shut down in YSTs. In pYSTs versus germinomas, high activity of BMP and WNT signalling has already been shown.^47^, ^48^, ^49^ So, activation of WNT, BMP and MAPK signalling, while the pluripotency network becomes inactivated are important steps in p/aYST formation or maintenance.

Furthermore, metallothioneins, chemokine receptors / ligands and HLA molecules become down‐regulated in YSTa versus ECs (Figure 4B). It remains elusive, if deregulations in these pathways are directly linked to activation of the FOXA2 axis or occur independently (Figure 4B).

We found that many of these key factors and driver processes might be controlled by DNA methylation and microRNAs (Figure 4B). During aYST formation, DNA methylation seems to be involved in silencing EC‐associated genes, especially pluripotency and PGC genes, such as *NANOG*, *BCAT1*, *DPPA4* and *DND1* (Figure 4B). In line with this finding, we already demonstrated that *NANOG* expression is regulated by DNA methylation in GCTs, with *NANOG*‐negative aYSTs harbouring with 70% the highest levels of *NANOG* promotor DNA methylation of all analysed GCT tissues (3.8% in seminomas, 6% in ECs, 66% teratomas and 62% choriocarcinomas).^50^

In this study, we highlight FOXA2 as a promising biomarker able to detect p/aYSTs with high specificity and to distinguish YST components from other GCT entities. Recently, another factor from the hepatocyte nuclear factor family (HNF1b) has been highlighted as a YST biomarker, further demonstrating the high similarity between YSTs and liver cells and suggesting that combining FOXA2 (HNF3b) and HNF1b to detect YSTs with high specificity in pathological routine diagnostics seems reasonable.^51^ Additionally, FOXA2 was sensitive enough to detect few YST cells in bulk tumour masses, like classical seminomas with elevated serum AFP levels, which has an important implication for clinical use; i. e. if FOXA2 is detectable in a patient's tumour sample, early more aggressive treatment may be recommended before YST outgrowth renders these progressive treatment‐resistant GCTs incurable.

## CONFLICT OF INTEREST

The authors confirm that there are neither conflicts of interest nor competing interests.

## AUTHOR CONTRIBUTIONS

**Wasco Wruck:** Data curation (equal); Formal analysis (equal); Investigation (equal); Software (equal); Visualization (equal). **Felix Bremmer:** Data curation (equal); Formal analysis (equal); Funding acquisition (equal); Investigation (equal); Methodology (equal); Visualization (equal); Writing‐review & editing (equal). **Mara Kotthoff:** Formal analysis (equal); Investigation (equal); Methodology (equal); Validation (equal); Visualization (equal). **Margaretha Skowron:** Formal analysis (equal); Investigation (equal); Methodology (equal); Supervision (equal); Writing‐review & editing (equal). **Alexander Fichtner:** Formal analysis; Investigation; Methodology; Validation; Visualization. **Stefan Schönberger:** Methodology; Resources (equal); Validation; Writing‐review & editing. **Gabriele Calaminus:** Resources (equal); Validation; Writing‐review & editing. **Christian Vokuhl:** Resources (equal); Validation; Writing‐review & editing. **David Pfister:** Resources (equal); Validation; Writing‐review & editing. **Axel Heidenreich:** Resources (equal); Validation; Writing‐review & editing. **Peter Albers:** Resources (equal); Validation; Writing‐review & editing. **James Adjaye:** Conceptualization (equal); Project administration (equal); Resources (equal); Supervision (equal). **Daniel Nettersheim:** Conceptualization (lead); Formal analysis (equal); Funding acquisition (lead); Investigation (equal); Methodology (equal); Project administration (lead); Resources (lead); Supervision (lead); Visualization (lead); Writing‐original draft (lead); Writing‐review & editing (equal).

## Ethical approval

The ethics committee (EC) of the Heinrich Heine University Düsseldorf (EC‐HHU‐D) raised no concerns about utilizing GCT cell lines for in vitro experiments (vote 2018‐178). All type I GCTs were cryopreserved and anonymized during the paediatric GCT MAKEI 96‐study^52^ and provided by the MAKEI 96‐biobank with no concerns raised by EC‐HHU‐D about analysing these samples (votes 837 and 2019‐822). Ethical approval for using the type II GCTs in the present study was obtained from the EC of the University Medical Centre Göttingen (vote 18/2/16).

## Supporting information

Figure S1

Figure S2

Figure S3

Table S1

Table S2

Table S3

Data S1

## Data Availability

Transcriptome data from YSTs and ECs were downloaded from the ‘National Center for Biotechnology Information Gene expression omnibus’ (NCBI GEO).^53^ In order to minimize technical variation, the meta‐analysis comprises only data sets generated on the Affymetrix Human Genome U133A platform (GSE3218, GSE10783, GSE7332, GSE8481, GSE10615). Gene expression, MicroRNA, DNA methylation and mutational burden data were extracted from the TCGA cohorts ‘Testicular GCT’, ‘Liver Hepatocellular Carcinoma’ and ‘Broad Cancer Cell Line Encyclopedia’. Histone‐chromatin‐immunoprecipitation‐sequencing (Histone‐ChIP‐seq) data sets (ENCSR000EXA/B/C/D/E/F/EWZ; ENCSR000AMB/AMD/AME/AOL/APV/ATD, ENCSR575RRX) were extracted from the ‘Encyclopedia of DNA elements’ (ENCODE) project.
