# Supplementary figures and images for: The pioneer and differentiation factor FOXA2 is a key driver of yolk‐sac tumour formation and a new biomarker for paediatric and adult yolk‐sac tumours
Source: J Cell Mol Med. 2021 Jan 14;25(3):1394–405. doi: 10.1111/jcmm.16222 (PMC7875904; doi:10.1111/jcmm.16222)

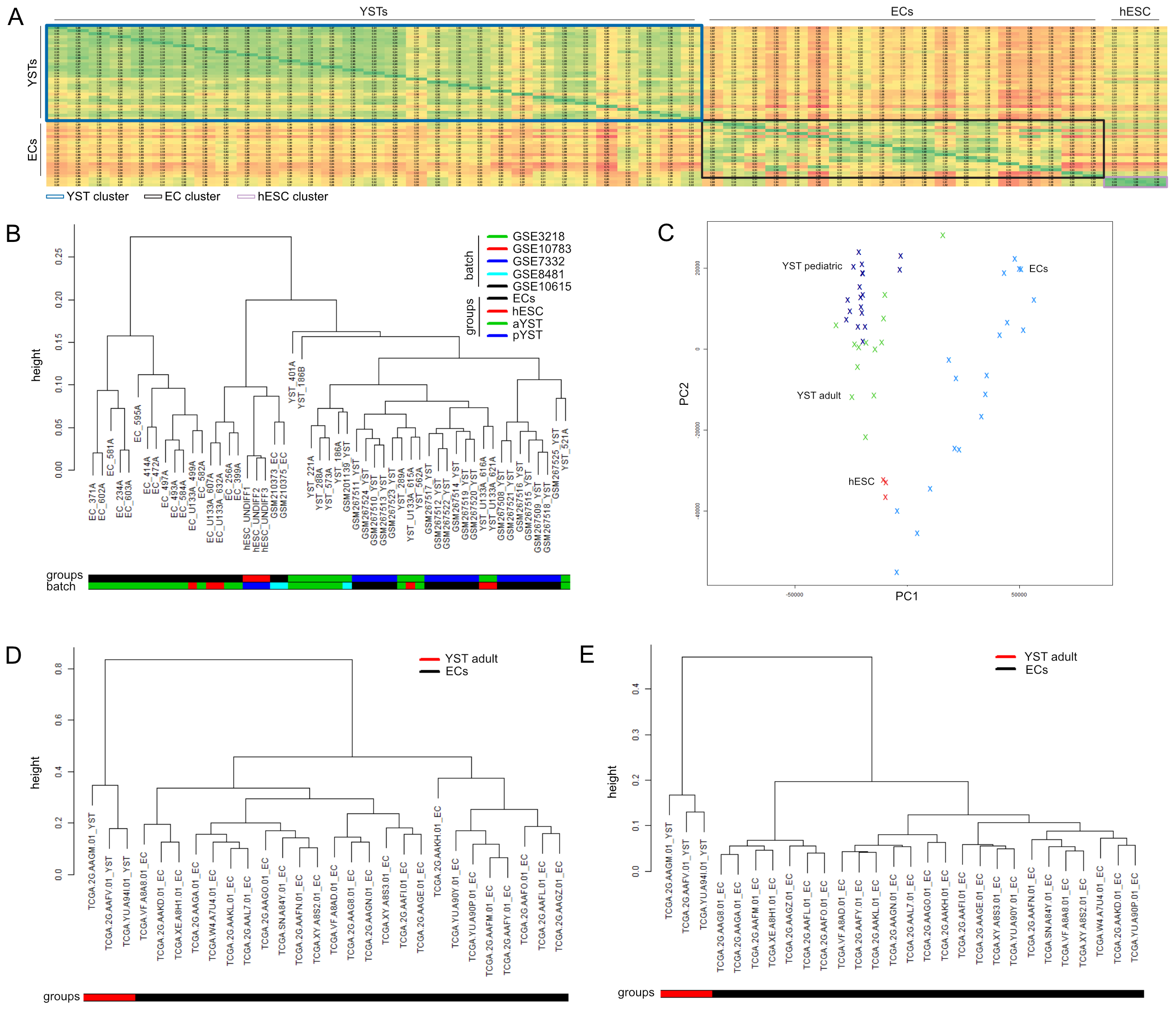

Supplement: Supplementary file 1 — Figure S1 [file JCMM-25-1394-s001.tiff]

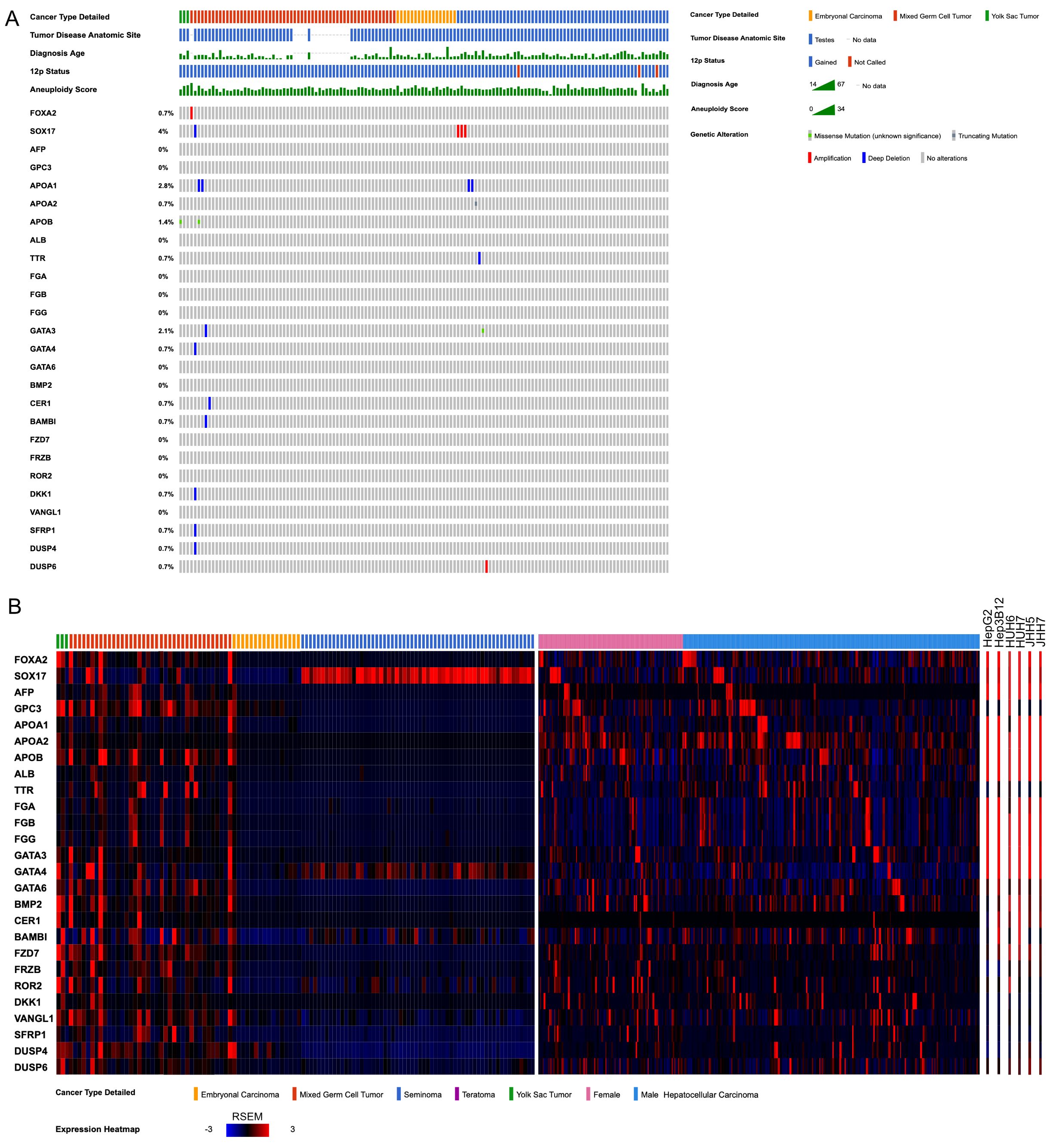

Supplement: Supplementary file 2 — Figure S2 [file JCMM-25-1394-s002.tiff]

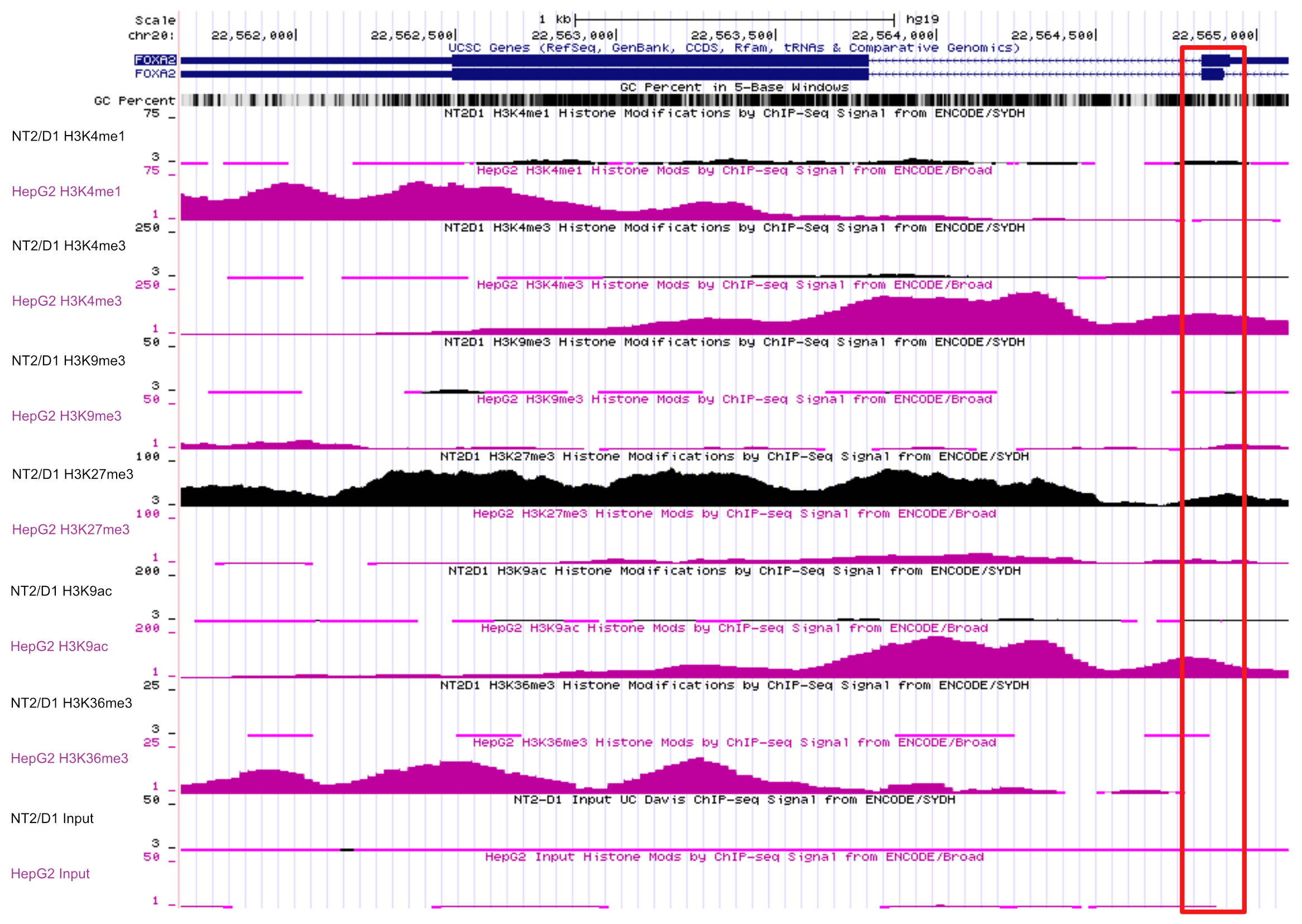

Supplement: Supplementary file 3 — Figure S3 [file JCMM-25-1394-s003.tiff]
